# Bifurcation kinetics of drug uptake by Gram-negative bacteria

**DOI:** 10.1371/journal.pone.0184671

**Published:** 2017-09-19

**Authors:** David A. Westfall, Ganesh Krishnamoorthy, David Wolloscheck, Rupa Sarkar, Helen I. Zgurskaya, Valentin V. Rybenkov

**Affiliations:** Department of Chemistry and Biochemistry, University of Oklahoma, Stephenson Parkway, Norman, OK, United States of America; Emory University School of Medicine, UNITED STATES

## Abstract

Cell envelopes of many bacteria consist of two membranes studded with efflux transporters. Such organization protects bacteria from the environment and gives rise to multidrug resistance. We report a kinetic model that accurately describes the permeation properties of this system. The model predicts complex non-linear patterns of drug uptake complete with a bifurcation, which recapitulate the known experimental anomalies. We introduce two kinetic parameters, the efflux and barrier constants, which replace those of Michaelis and Menten for trans-envelope transport. Both compound permeation and efflux display transitions, which delineate regimes of efficient and inefficient efflux. The first transition is related to saturation of the transporter by the compound and the second one behaves as a bifurcation and involves saturation of the outer membrane barrier. The bifurcation was experimentally observed in live bacteria. We further found that active efflux of a drug can be orders of magnitude faster than its diffusion into a cell and that the efficacy of a drug depends both on its transport properties and therapeutic potency. This analysis reveals novel physical principles in the behavior of the cellular envelope, creates a framework for quantification of small molecule permeation into bacteria, and should invigorate structure-activity studies of novel antibiotics.

## Introduction

Gram-negative pathogens that are resistant to almost all currently available antibiotics are rapidly spreading in clinics [1]. New antibiotics targeting these pathogens are urgently needed. Several decades of antibiotic discovery identified the permeability barrier as the major hurdle in the development of new therapeutics against Gram-negative pathogens [2–4]. Significant efforts are presently directed at fundamental understanding of the permeability properties of the outer membrane and at finding correlations between physicochemical properties of compounds and their permeation across cell envelopes [5].

A major impediment to these efforts is the lack of a quantitative model that would match the complexity of the system and adequately describe drug accumulation in bacteria [2, 5, 6]. The current views hold that small molecules penetrate into bacterial cells via passive diffusion and, therefore, obey Fick’s laws [7, 8]. The ensuing models, however, fail to produce predictive structure-activity relationships for compound permeation into the cell [9, 10], explain the frequently observed sigmoidal uptake curves [11, 12], or find correlations between permeation of drugs and their potency [11, 13]. The attempts to refine the model and incorporate drug efflux have been done only numerically or in approximation and failed to detect the non-linearity inherent to this system [7, 11]. We describe and validate here a model that recuperates the observed experimental abnormalities and offer a practical analytical solution to the problem.

**Fig 1:**
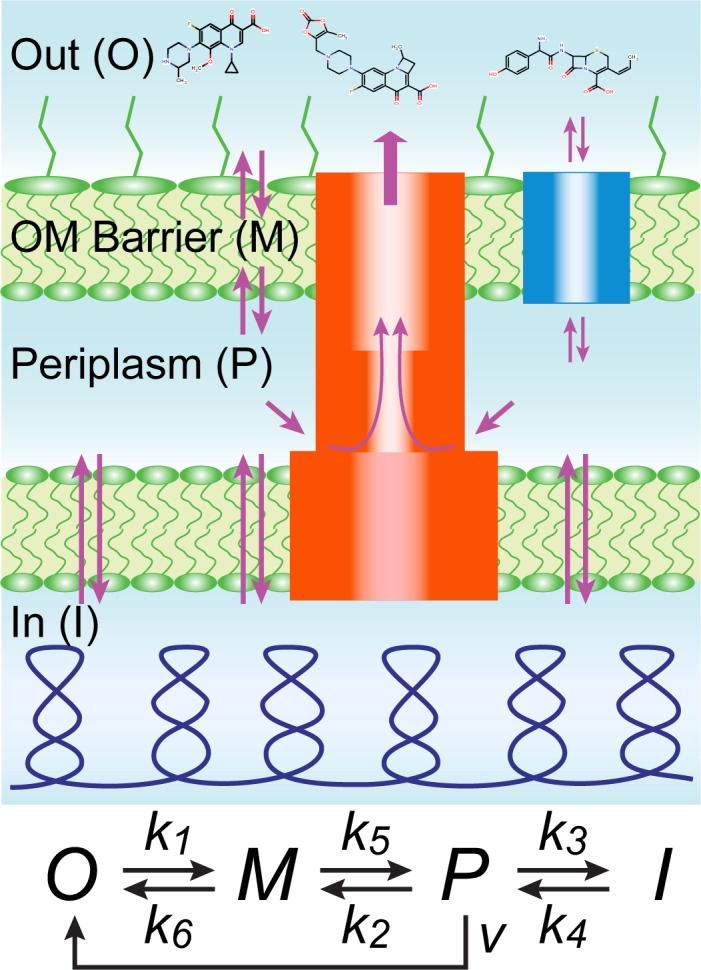
Major fluxes into bacteria, including passive diffusion across the inner and outer membranes, facilitated diffusion through porins and channels (blue), and active efflux by multidrug transporters, such as AcrAB-TolC of *E*. *coli* (red). Labeled are the four main compartments considered in the kinetic scheme including the outside (*O*), the outer membrane barrier (*M*), the periplasm (*P*), and internal space (*I*). The kinetic scheme summarizes these processes (see text for details).

Cell envelopes of Gram-negative bacteria consist of two lipid membranes with different physicochemical structures and functions. The outer membrane is an asymmetric bilayer of lipopolysaccharides (LPS) and phospholipids, into which non-specific porins and specialized uptake channels are embedded [14]. This membrane creates a formidable permeability barrier for both hydrophilic and lipophilic molecules [7, 15–18]. Compounds that permeate through the outer membrane encounter the periplasm and the inner membrane together with poly-specific efflux pumps that actively expel a variety of compounds and further reduce their intracellular concentrations [19–21]. The structural signature of the major efflux pumps is the three-component protein assembly spanning the two membranes and the periplasm [22, 23]. AcrAB-TolC from *Escherichia coli* is the best studied example of such pumps that expel multiple antibiotics from the periplasm across the outer membrane and create a synergistic permeability barrier (Fig 1).

The permeability properties of the outer membrane are largely defined by general porins with an exclusion limit of about 600 Da in *E*. *coli* [4, 12, 24–26]. Most hydrophilic and amphiphilic antibiotics reach the periplasm through the porins, whereas larger antibiotics, for example erythromycin or novobiocin are believed to cross the outer membrane through the LPS-phospholipid bilayer [14, 17, 27, 28]. Porins lack specificity and interact with diffusing solutes only with low affinities. The measured residence times of small molecules within reconstituted porins are in sub-millisecond range [29]. This indicates that the flux through a given channel is subject to saturation. However, no such saturation was observed for uptake of beta-lactam antibiotics into live *E*. *coli* cells [30].

Several attempts have been made to construct a quantitative model of drug permeation into the cell. It became clear that individual transporters well conform to the Michaelis-Menten kinetics [11, 12, 31–33]. However, integrating Michaelis-Menten drug efflux with transmembrane diffusion proved challenging, presumably because the resulting differential equations cannot be solved analytically [7, 11]. Moreover, experimental uptake studies produced at times sigmoidal concentration dependencies, which could not be explained by the available models [11, 12]. We describe, analytically solve and validate here a model that recuperates various experimental abnormalities and discuss implications of this analysis.

## Results

### The model

Fig 1 summarizes the current view on drug permeation into Gram-negative pathogens. Three main factors limit the intracellular accumulation of antibiotics in bacteria: the highly impermeable outer membrane barrier, the inner membrane, and active efflux of compounds from the periplasm into the external medium by multidrug transporters [4, 17, 34]. Here we analyzed for the first time how all three factors function together. As a key innovation, we explicitly model drug interaction with the outer membrane to recognize that many compounds can only slowly cross it.

Fig 1 shows a kinetic scheme that approximates the process. In this scheme, *O*, *M*, *P* and *I* are the drug concentrations in the external medium, outer membrane, periplasm and cytoplasm, respectively; *k*_*1*_ through *k*_*6*_ are rate constants for drug diffusion across the compartments, and *v* is the rate of active drug efflux. Conforming to previous studies [11, 31, 35], we postulate that active drug efflux follows Michaelis-Menten kinetics, *v = V∙P/(Km+P)* with the maximal transporter flux *V* and Michaelis constant *Km*. To account for the slow diffusion of compounds across the outer membrane, we treat the outer membrane barrier as a separate compartment and assume that it has a limited number of drug binding sites, *M*_*0*_, which can be saturated at high external drug concentrations. Although frequently overlooked, this condition applies both to compounds that diffuse into cells through the lipid bilayer and transmembrane channels since even the latter show a detectable delay during translocation [18, 36, 37]. This condition proved to be essential for fitting our experimental data (see below). The rate of crossing the barrier would be proportional then to the fraction of unoccupied sites on the barrier, (1-*M/M*_*0*_). The model also envisions that a drug might have binding partners inside the cell and, therefore, no equality is expected between *k*_*1*_ and *k*_*2*_ and between *k*_*3*_ and *k*_*4*_.

The reaction in Fig 1 is equivalent to the following set of differential equations:

$$\frac{d}{dt}M=k_{1}O\left(1-\frac{M}{M_{0}}\right)-(k_{5}+k_{6})M+k_{2}P\left(1-\frac{M}{M_{0}}\right),$$ (1A)

$$\frac{d}{dt}P=k_{5}M-k_{2}P\left(1-\frac{M}{M_{0}}\right)-V\frac{P}{Km+P}-k_{3}P+k_{4}I,$$ (1B)

$$\frac{d}{dt}I=k_{3}P-k_{4}I.$$ (1C)

We focus on the practically important limit, when the external volume vastly exceeds the volume of bacteria, and therefore, the external drug concentration remains constant throughout the experiment, *O* = const. In the absence of active efflux and saturation (i.e. *V* = 0, *M*_*0*_ = ∞), the well-known solution to Eq 1 is given by a triple-exponential decay, with the rates equal to the eigenvalues of the system. With active efflux, the resulting differential equations are non-linear and require numerical integration. The system, however, can be solved at two extremes important for practical applications: steady state drug accumulation levels and initial rates.

### Steady state drug uptake levels

Under steady state conditions, all derivatives in Eq 1 reduce to zero, and the solution to the system is given by a quadratic equation (see Methods for derivation):

$$P^{2}+P∙\left(\frac{Km}{1+B}∙(1+K_{E})-\frac{1-B}{1+B}∙X_{p}\right)-\frac{Km}{1+B}∙X_{p}=0,$$ (2A)

$$I=k_{3}/k_{4}∙P,$$ (2B)

where *X*_*p*_ *= k*_*1*_*∙O/k*_*2*_ is the equilibrium concentration of compounds in the periplasm in the absence of active efflux, *K*_*E*_ *= V/(Km∙0*.*5k*_*2*_*)* is the efflux constant, *B = V/F* is the barrier factor, and *F = k*_*5*_*M*_*0*_ is the maximal flux across the outer membrane. The efflux constant relates the thermodynamic efficiency of active transporters, *V/Km*, and the diffusion rate across the membrane, *0*.*5k*_*2*_ (see also Eq 10). Thus, the efflux and barrier constants relate the active and passive fluxes across the membrane at the limiting and saturating drug concentrations, respectively. Eq 2A has a single positive root (Eq 9). Because Eq 9 has a characteristic structure, we introduced a function that yields the desired root to equations of this kind. In general, this *R*-function has four arguments and is defined as:

$$R(X,a,b,c)=\frac{1}{2}∙\left[-a(1+b)+cX+\sqrt{{\left(a(1+b)-cX\right)}^{2}+4aX)}\right].$$ (3A)

This function provides the positive root (for positive *X*) to a second order polynomial with the structure:

$$P^{2}+P∙(a(1+b)-cX)-aX=0.$$ (3B)

Given these definitions, the solution to Eq 2 can be presented as:

$$P=R\left(X_{p},\frac{Km}{1+B},K_{E},\frac{1-B}{1+B}\right).$$ (4)

**Fig 2:**
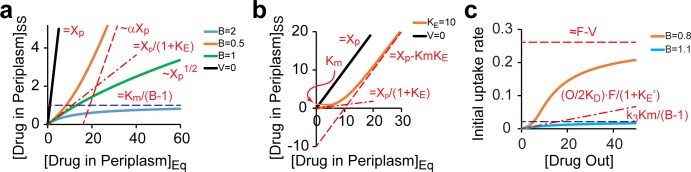
Characteristic regimes of drug accumulation in bacteria. (**a**). Steady state (ss) drug levels in the periplasm plotted against the equilibrium (eq) drug concentration (*X*_*p*_), which would be observed in the absence of active fluxes. Note that *X*_*p*_ is proportional to the external drug concentration, *X*_*p*_ *= k*_*1*_*O/k*_*2*_. Solutions to Eq 2 are shown for cells without active drug efflux (*V = 0*), or with the indicated values of the barrier constant *B*. Asymptotic behavior of the plots (Eq 11) is shown with dashed lines together with the underlying equations. The variables are defined in Eq 2; α = (1-*B*)/(1+*B*). Note the existence of two regimes, of efficient (*B>1*) and inefficient (*B<1*) efflux. (**b**). The relationship between the steady state and equilibrium drug concentrations in the periplasm of cells with active drug efflux (*K*_*E*_ > 1) but no outer membrane barrier (*B* = 0). The transition between regimes of efficient and inefficient efflux occurs when the periplasmic drug concentration reaches the transporter’s Michaelis constant *Km*. (**c**). The initial rate of drug accumulation in the cytoplasm as a function of its external concentration. Dashed lines mark the asymptotes to the plots.

When applied to drug uptake, the *R*-function describes a monotonic relationship between the steady state and equilibrium concentrations of the drug (*P* versus *X*_*p*_) with the other three arguments acting as parameters that define the shape of the dependence (S1 Fig). The solution shown in Eq 4 displays different patterns depending on the values of *B* and *K*_*E*_ (Fig 2A). Two transitions between these patterns can be observed, one related to saturation of the efflux transporter and the other to saturation of the outer membrane barrier.

At low drug concentrations (*P << Km*, i.e. when *X*_*p*_ →0), the intracellular and external drug concentrations are linearly related (as are the steady state and equilibrium concentrations), so that *P = Xp / (1+K*_*E*_*)* (Fig 2A). Thus, the steady state drug concentration is reduced by a factor of *1+K*_*E*_ compared to the equilibrium. In this sense, the efflux constant serves as a measure of thermodynamic efficiency of a transporter that acts against diffusion. At higher drug levels (*P > Km*), the transporter is saturated (*v ≈ V*), and the equilibrium is shifted by only a constant value, *ΔP* = *V/(0*.*5k*_*2*_*) ≡ Km∙K*_*E*_ (Fig 2B).

The second transition is controlled by the value of *B*, which compares maximal possible fluxes across the barrier and via the transporter. Importantly, the aforementioned pattern can only be observed when *B < 1*, and even then with a reduced slope of the *P* vs. *Xp* dependence (Fig 2A). When *B* is greater than 1, the barrier becomes saturated before the transporter does, and the intracellular drug concentration plateaus out at *Km/(B-1)* (Fig 2A). Such compounds can never exceed the plateau level unless the barrier or efflux are breached. Therefore, this regime encompasses conditions of efficient efflux. The transition between the two regimes occurs at *B =* 1, when the intracellular and external drug concentrations are parabolically related (Fig 2A). The condition *B* = 1 defines a bifurcation point in the solution whereat its functional and asymptotic behavior changes. Of note, it can be shown that the switch from the convex to concave shape of the function occurs at slightly smaller values of *B*, *B = K*_*E*_*/(2+K*_*E*_*)*. Importantly, the system has a single solution in the positive quadrant (positive *P* and *X*_*p*_) at all values of *B*, and only the shape of the solution and its range change. In this sense, the bifurcation displayed by this system differs from the conventional ones, which describe changes in the number of solutions and their stability.

### Initial rates of drug accumulation

Compounds with slow accumulation rates might never reach the steady state during the course of experiments. In such cases, initial rates are typically measured. The initial rate of drug accumulation in the cytoplasm, *V*_*I*_, can be calculated as *V*_*I*_ *= k*_*3*_*∙P*_*b*_, where *P*_*b*_ is the burst size of *P*. The solution for *P*_*b*_ can again be reduced to a quadratic equation (Eq 14 in Methods). Combining Eq 14 and the definition of the *R*-function, the solution for initial rates will be given by:

$$V_{I}=k_{3}∙R\left(\frac{F\varphi }{k_{3}+k_{2}(1-\varphi )},Km,\frac{V}{Km(k_{3}+k_{2}(1-\varphi ))},1\right),$$ (5)

where *φ = O/(O+2K*_*D*_*)* is the degree of saturation of the barrier. Calculations based on Eq 5 are in excellent agreement with the results of direct numeric modeling of the reaction in Eq 1 (S2 Fig).

Similar to the steady state levels, the initial rates display two distinct patterns defined by the value of *B*. Unlike with the steady state, the initial rates reach saturation in both regimes (Fig 2C). This saturation is caused by the limited capacity of the outer membrane barrier, which cannot allow infinitely high fluxes. For conditions of efficient efflux (*B > 1*), the initial rate plateaus out at *k*_*3*_*Km/(B-1)*. When the flux through the barrier can overcome the transporter (*B<1*), the dependence is sigmoidal with the plateau at approximately *F-V* (Fig 2C). Notably, the transition between the two regimes is sharp and can lead to dramatic changes in drug permeation in response to even fractional changes in the value of *B* (Fig 2C).

### Experimental validation of the model

To validate the model, we examined uptake of bisbenzimide (Hoechst 33342), a DNA binding drug that inhibits topoisomerases [38]. Hoechst fluorescence increases 134- and 31-fold, respectively, upon binding to DNA or lipids (S3 Fig). This compound diffuses slowly between leaflets of the cytoplasmic membrane [39] and is pumped out from membranes by multidrug efflux transporters [40, 41]. These features make it a popular compound for drug uptake studies [42–44].

**Fig 3:**
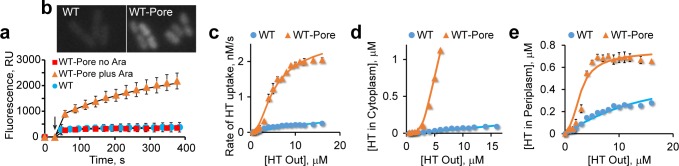
Accumulation of Hoechst 33342 (HT) in *Escherichia coli*. (**a**). Time courses of Hoechst accumulation (±SD; *n* = 4) in WT and WT-Pore cells grown without and with 0.1% arabinose to induce the expression of the pore. The data were fit to a burst- single exponential decay, *I* = *A1* + *A2*∙(1-exp(-*k*_*2*_*t*)), where *A1* describes the fast initial step, and the slow step reflects subsequent rise in fluorescence (see Methods for details). Arrow marks the moment when cells were mixed with 4 μM Hoechst (**b**). Fluorescence microscopy analysis of Hoechst uptake by WT and WT-Pore cells grown with 0.1% arabinose, following incubation with 1 μM Hoechst for 1 min. (**c**). Initial rates of Hoechst accumulation in WT and WT-Pore cells grown with 0.1% arabinose during the slow phase shown in panel **a** and S4 Fig. The data were fit to Eq 5. (**d**). Steady state concentrations of Hoechst in the cytoplasm fit to Eq 4. (**e**). Steady state concentrations of Hoechst in the periplasm.

Exponential *Escherichia coli* BW25113 (WT) cells were collected at OD_600_ of 1, suspended in buffered glucose solution, supplemented with Hoechst, and its fluorescence followed. Following the initial burst, caused by binding to cell membranes, Hoechst fluorescence steadily increased (Fig 3A and 3B). The initial rates displayed a hyperbolic concentration dependence (Fig 3C), which is in full accord with Eq 5. In contrast, a linear concentration dependence would be expected should the system be governed by the Fick’s law of diffusion or the Michaelis-Menten behavior of the transporter. This result, therefore, is a strong indication that the model adequately describes drug uptake by bacteria. Furthermore, these data reveal that the barrier constant *B* for Hoechst is about equal to 1 or greater in the wild type *E*. *coli*. In other words, the dye penetrates into *E*. *coli* via facilitated diffusion.

To confirm this result, we examined Hoechst uptake by the otherwise isogenic WT-Pore cells, which encode FhuA* protein under the control of an arabinose inducible promoter. FhuA* is a genetically modified channel protein that, when expressed, creates an open 2.4 nm pore [45] in the outer membrane and, thereby, increases the rate of drug permeation across the membrane [46]. WT-Pore cells, therefore, are expected to have a lower *B*-factor compared to WT cells and should display a different kinetic behavior.

As expected, WT-Pore cells were indistinguishable from WT in the absence of the arabinose inducer. However, the initial rates of Hoechst uptake by WT-Pore cells displayed a sigmoidal concentration dependence in the presence of arabinose (Fig 3C). This is, again, in full accord with Eq 5. Moreover, the steady state level of the cytoplasmic, DNA bound Hoechst (Fig 3D) and the burst level of the periplasmic, membrane bound Hoechst (Fig 3E) were also fully consistent with the predictions of the model.

All three data sets could be fit to the same set of model parameters over the entire concentration range, simultaneously for WT and WT-Pore cells (see Methods). This agreement strongly argues that the model correctly recuperates the main features of Hoechst uptake by bacteria. According to the fitting, the flux of Hoechst across the outer membrane increased by 33% following induction of FhuA*, thereby reducing *B* from 0.98 to 0.74 (Fig 3C). The efflux constant *K*_*E*_ for the wild type strain was estimated as 420. Thus, multidrug transporters in *E*. *coli* remove Hoechst a staggering 420 fold faster than it can diffuse into the cell. The same set of model parameters (with adjusted *B*) could describe Hoechst uptake by WT-Pore cells grown at various concentrations of arabinose, which results in graduated expression of FhuA* (S5 Fig).

### Relationship between uptake and growth inhibition

**Fig 4:**
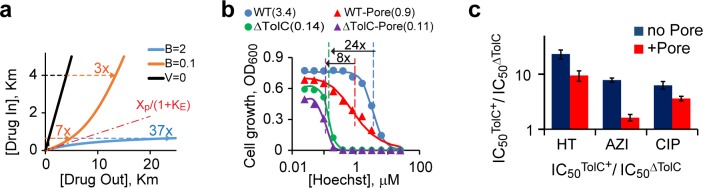
Contributions of active drug efflux and the outer membrane barrier to drug susceptibility of a bacterium. (**a**) Effects of the inactivation of drug efflux are modeled for bacteria with efficient (*B > 1*) and inefficient (*B < 1*) efflux. Drug concentrations are measured in the units of *Km*, *K*_*E*_ equals 10 for all compounds. Depending on the intracellular inhibition constant of a compound, *K*_*I*_, inactivation of efflux can reduce its observed inhibitory concentration 3-, 7- or 37-fold, respectively, in the case of inefficient (*B* < 1, *K*_*I*_ > *Km*), partially efficient (*B* < 1, *K*_*I*_ < *Km*) or efficient (*B* > 1, *K*_*I*_ < *Km*) efflux. (**b**). Growth inhibition of bacteria with defects in multidrug efflux or membrane permeability by Hoechst. The IC_50_ values (in μM) are shown in parenthesis. (**c**). The ratio of IC_50_s of azithromycin (AZI), ciprofloxacin (CIP) and Hoechst (HT) for TolC^+^ and ΔTolC cells measured in the absence (no Pore) or presence (+Pore) of overproduced FhuA* (±SD; *n* ≥ 2) plotted on a logarithmic scale.

As a final test of the model, we compared the potency of Hoechst as a bacterial growth inhibitor in strains with altered cell envelope. We employed a set of *E*. *coli* strains that differ in the presence of the FhuA* pore and the TolC channel, which is required for several *E*. *coli* efflux transporters [46]. The above analysis predicts that the contribution of efflux to bacterial drug resistance depends not only on the transport properties of the compound but also its potency. When the transporter is far from saturations (i.e. *P < Km*) are the conditions of efficient efflux. The intracellular concentration of such compounds is dramatically reduced, by a factor of *1+K*_*E*_, compared to that in the medium. Inactivation of drug efflux in such cells should increase drug susceptibility by *1+K*_*E*_ fold. Conversely, the steady state level of a compound approaches its equilibrium value when *P > Km*. For such compounds, the contribution of efflux to drug resistance is only minimal (Fig 4A). The latter conditions, however, can only be achieved when *B* is less than 1. When *B* is greater than 1, the transporter is below saturation even at high external levels of the drug, and the barrier amplifies the protective action of the pump (Fig 4A). Consistent with these expectations, we found that inactivation of TolC-dependent efflux transporters decreases the inhibitory concentration of Hoechst, IC_50_, by 24-fold in the WT but only 8-fold in the WT-Pore cells (Fig 4B). Similarly, the deletion of TolC had a stronger effect on bacterial susceptibility to antibiotics azithromycin and ciprofloxacin in the absence of the pore than in its presence (*p* < 0.01, Fig 4C).

## Discussion

The model described here provides a realistic account of drug uptake by Gram-negative bacteria and should find broad applications in the antibiotic discovery and development and the analysis of cell interaction with the environment. The elements of the model are firmly rooted in the current knowledge of drug permeation but their integration has been achieved for the first time. The main obstacle has been in the non-linear character of the underlying differential equations which can only be numerically integrated [7]. We show here that this problem can be solved analytically in the steady-state approximation. We describe the solution in terms of the *R*-function, which provides the positive root to quadratic equations of a specific structure (Eq 3). We found that the *R*-function provides a solution to a range of problems of this class (Eqs 4 and 5).

The drug uptake patterns were non-linear, contained bifurcations and did not conform to the Fick’s Law of diffusion or Michaelis-Menten kinetics (although both were integrated into the model). The behavior of the system could be described in terms of two new kinetic parameters, the efflux constant *K*_*E*_ and the barrier constant *B*. The two parameters relate the efficiencies of active efflux and passive diffusion, as observed, respectively, at low and high drug concentrations, and describe different aspects of drug uptake. The efflux constant reports by how much the intracellular concentration of the drug is reduced compared to thermodynamic equilibrium, under conditions when the transporter operates below saturation. This number proved to be remarkably large and was estimated as 420 for Hoechst. The barrier constant describes what happens to the system at high drug concentrations and discriminates conditions of efficient and semi-efficient efflux. When *B* is smaller than 1, the transporter could be saturated by high external concentrations of the drug; this would not happen when *B* is greater than 1.

Importantly, the model does not make any assumptions about the molecular organization of the outer membrane barrier. It simply recognizes that the flux through the membrane is reduced compared to free diffusion, if only because the combined surface area of the porins is smaller than the surface of the cell. Only the total flux through the barrier, *F*, shows up in the final equations, but not its specific implementation. For compounds that cross the outer membrane barrier without binding it, the maximal flux is limited by the rate of diffusion through the combined area of the pores. In the case of Hoechst uptake, postulating the barrier was dictated by the experiment, which revealed saturation in the accumulation rates of the dye (Fig 3). Such saturation cannot be explained by active efflux or limited availability of the intracellular target but implies saturation of the flux across one of the partitions.

Rather counterintuitively, the model predicts that drug efflux does not provide equal protection against all compounds. Instead, its effect depends on potency of the drug. In general, more toxic compounds are better protected against than compounds with high inhibition constant (Fig 4A). Moreover, even small variations in the efficiency of the barrier can have dramatic effects on antibiotic susceptibility (Fig 4). These features might explain why the measured drug permeation rates show poor correlation with their potency [13, 47] and how a small increase in the activity of an efflux transporter can markedly increase drug resistance of a bacterium. The use of the model offers a plausible path around this perplexing observation and should invigorate drug permeation studies aimed at the discovery of novel antimicrobials.

The synergistic interaction between active efflux and the outer membrane conveys new properties to the cell. In certain cases, when *B* is greater than one, the cell envelope behaves as a rigid partition that effectively blocks access to the cell for a variety of external chemicals. This occurs even though the membrane itself might remain highly permeable to these chemicals on a molecular level. Thus, the collective behavior of the individual components gives rise to a novel quality that likens the system to a macroscopic object. Indeed, similar to a hull of a boat, cellular membrane remains virtually impermeable to external compounds until its integrity is breached (Figs 2 and 3). In this sense, the implications of the model transcend its biological utility. A special formalism needs to be devised to describe such systems. We began creating such formalism by introducing the *R*-function and two new kinetic parameters, the flux and barrier constants. This approach provides means for delineating the contributions of the outer membrane and active drug efflux in activities of antimicrobial agents and will become indispensable in quantitative and empirical structure-activity studies of antibiotics targeting Gram-negative pathogens.

## Materials and methods

### Steady state concentrations

Under steady state conditions, Eq 1 reduces to a set of algebraic equations:

$$0=k_{1}O\left(1-\frac{M}{M_{0}}\right)-(k_{5}+k_{6})M+k_{2}P\left(1-\frac{M}{M_{0}}\right),$$ (6A)

$$0=k_{5}M-k_{2}P\left(1-\frac{M}{M_{0}}\right)-V\frac{P}{Km+P}-k_{3}P+k_{4}I,$$ (6B)

$$0=k_{3}P-k_{4}I.$$ (6C)

Because binding and dissociation of drugs to the barrier are not accompanied by dissipation of the free energy, we can further postulate that *k*_*5*_ *= k*_*6*_. This condition indicates that the state *M* is the same as the transition state during translocation of the compound across the outer membrane, which is the state at the top of its free energy profile on a path from *O* to *P*. Such a point always exists unless the compound crosses the membrane via active transport. This state is not the same as any of experimentally observable stable complexes of the compound with the membrane, for which *k*_*5*_ and *k*_*6*_ do have to be equal. Eq 6 can be readily rearranged to the following form:

$$M=M_{0}∙\frac{k_{1}O+k_{2}P}{{k_{1}O+k_{2}P+(k}_{5}+k_{6})M_{0}},$$ (7A)

$$k_{5}M-k_{2}P(1-\frac{M}{M_{0}})=V∙\frac{P}{Km+P},$$ (7B)

$$I=\frac{k_{3}}{k_{4}}∙P.$$ (7C)

Eqs 7A and 7C can be immediately solved once the answer to Eq 7B is known. To this end, *M* was substituted from Eq 7A into Eq 7B, and the latter further rearranged to yield:

$$\left(1+\frac{V}{{k_{6}M}_{0}}\right)P^{2}+P\left(Km+\frac{V}{k_{2}}∙\frac{k_{5}+k_{6}}{k_{6}}-\frac{k_{1}O}{k_{2}}∙(\frac{k_{5}}{k_{6}}-\frac{V}{k_{6}M_{0}})\right)-Km∙\frac{k_{5}}{k_{6}}∙\frac{k_{1}O}{k_{2}}=0.$$ (8A)

Recalling that *k*_*5*_ *= k*_*6*_ in the absence of energy fluxes on the outer membrane, and introducing new variables *X*_*p*_ *= k*_*1*_*O/k*_*2*_, *K*_*E*_ *= (V/Km)∙k*_*2*_^*-1*^*∙(k*_*5*_*+k*_*6*_*)/k*_*6*_, and *B = V/(k*_*6*_*M*_*0*_*)*, we can rewrite the equation to the form equivalent to Eq 2:

$${(1+B)∙P}^{2}+P∙(Km∙(1+K_{E})-(1-B)∙X_{p})-Km∙X_{p}=0.$$ (8B)

This quadratic equation has a single positive root given by:

$$P=\frac{0.5}{1+B}∙\left[-Km(K_{E}+1)+(1-B)X_{p}+\sqrt{{\left(Km(K_{E}+1)-(1-B)X_{p}\right)}^{2}+4KmX_{p}(1+B)}\right].$$ (9)

Eq 9 offers an explicit solution for the steady state drug concentration in the periplasm and, when combined with the Eq 6C, in the cytoplasm. Using the definition of the *R*-function (Eq 3), this solution can also be written in the form of Eq 4.

It is instructive to review the meaning of the introduced variables. *k*_*1*_*/k*_*2*_ is the equilibrium constant for the transfer of a compound from the external medium into the periplasm. Indeed, the equilibrium constant for this process equals *k*_*1*_*k*_*5*_*/k*_*2*_*k*_*6*_ = *k*_*1*_*/k*_*2*_, since *k*_*5*_ = *k*_*6*_. Therefore, *X*_*p*_ represents the equilibrium concentration of the compound in the periplasm provided there are no active fluxes. Thus, Eqs 9 and 5 have a trivial solution when *V = 0*, *P = X*_*p*_.

*k*_*6*_*/(k*_*5*_*+k*_*6*_*)* is the probability of the drug within the outer membrane barrier to dissociate into the medium rather than the periplasm, and, therefore, *k*_2_* = k_*2*_*∙k*_*6*_*/(k*_*5*_*+k*_*6*_*) = 0*.*5k*_*2*_ represents the rate of diffusion of the drugs from the periplasm into the medium. In this light, the definition of the efflux constant can be rewritten as:

$$K_{E}=\frac{V}{k_{2}^{*}∙Km}.$$ (10)

Since *V/Km* represents the microscopic rate constant of active efflux at low drug concentrations (*P < Km*), *K*_*E*_ relates the rates of drug efflux via the transporter and passive diffusion at low concentrations of the drug. The *B*-factor describes a similar ratio but for saturating drug concentrations, when *P > Km*.

The asymptotic behavior of Eq 9 was analyzed using its expansion into Taylor series. The results are summarized below and in Fig 2A.

$$P=\frac{X_{p}}{1+K_{E}},X_{p}\to 0,$$ (11A)

$$P=\frac{Km}{B-1},X_{p}\to \infty ,B>1,$$ (11B)

$$P=\frac{1-B}{1+B}∙X_{p}+\frac{Km}{1-B}-\frac{Km(K_{E}+1)}{1+B},X_{p}\to \infty ,B<1,$$ (11C)

$$P={\left(KmX_{p}\right)}^{1/2},X_{p}\to \infty ,B=1.$$ (11D)

### Initial rates

Numerical integration of Eq 1 was done using the home written Matlab program Efflux. An example of this analysis is shown in S2 Fig. The equation can also be solved analytically for initial rates if one takes advantage of the hierarchy of times often found in such systems. Here, we limit the analysis to compounds that have a binding target in the cytoplasm, *k*_*4*_ *<< k*_*3*_. For such systems, the steady state approximation can be applied to both transient intermediates *M* and *P* [48]. We further limit ourselves to systems with strong efflux, so that *k*_*2*_*P* << *k*_*5*_*M* at all times. Note that a similar result can be achieved if the relaxation rate for formation of *M*, *k*_*m*_ *= k*_*1*_*O/M*_*0*_*+k*_*5*_*+k*_*5*_, exceeds the relaxation rate for formation of *P*, *k*_*p*_ *≈ V/(Km+P)+k*_*2*_*+k*_*3*_.

Under such conditions, the accumulation of *M* is described by Eq 1A with *P* equal to zero. The completion of this phase results in a burst of *M* with the burst size, *M*_*b*_, given by

$$M_{b}=M_{0}∙\frac{k_{1}O}{{k_{1}O+(k}_{5}+k_{6})M_{0}}=M_{0}∙\frac{O}{O+2K_{D}},$$ (12)

where *K*_*D*_ *= 0*.*5M*_*0*_*∙(k*_*5*_*+k*_*6*_*)/k*_*1*_ *= k*_*6*_*M*_*0*_*/k*_*1*_ is the dissociation constant of the drug bound to the outer membrane barrier. Until the burst is complete, accumulation of the drug in the periplasm will be limited, resulting in a characteristic lag in the time course of *P* (S2A Fig).

The second event involves a build-up in *P* as described by Eq 1B with *I* = 0. This phase is complete when *dP/dt* declines to zero, which leads to the following equation for the burst size of *P*:

$$k_{5}M_{b}-k_{2}P\left(1-\frac{M_{b}}{M_{0}}\right)-V\frac{P}{Km+P}-k_{3}P=0.$$ (13)

Following the substitutions ϕ *= M*_*b*_*/M*_*0*_ and *F = k*_*5*_*M*_*0*_, Eq 13 can be recast to the form suggested in Eq 3B:

$${P_{b}}^{2}+P_{b}∙\left(Km+\frac{V}{k_{2}(1-\varphi )+k_{3}}-\frac{F\varphi }{k_{2}(1-\varphi )+k_{3}}\right)-Km∙\frac{F\varphi }{k_{2}(1-\varphi )+k_{3}}=0,$$ (14A)

$$V_{I}=k_{3}∙P_{b}.$$ (14B)

The slowest event in this process is the build-up of the cytoplasmic drug content towards its steady state level, which in turn creates diffusional fluxes back into the periplasm and thereby leads to the build-up of *P* and *M* from their burst levels to the steady state (S2A Fig). The initial rate for the drug accumulation in the cytoplasm can be calculated then according to Eq 14B, which is equivalent to Eq 5. As shown in S2B Fig, the initial rates calculated according to Eq 5 and obtained by numeric integration of Eq 1 are virtually identical. Asymptotic behavior of this solution is summarized in Fig 2C.

### Fitting Hoechst uptake data

The time courses of Hoechst uptake were fit to the burst-single exponential decay function *F* = *A*_*1*_+*A*_*2*_∙(1-exp(-*kt*)), where *A*_*1*_ and *A*_*2*_ describe the magnitude of the fast and slow steps, respectively, and *k* is the rate of the slow step. The fast and slow steps were attributed to Hoechst binding to the lipids (i.e. in the periplasm) and chromosomal DNA (i.e. in the cytoplasm), respectively. The best fit amplitudes were then converted into concentrations of the intracellular Hoechst using the measured emission coefficients for the Hoechst-lipid and Hoechst-DNA complexes (S3 Fig) and plotted as the experimental steady state Hoechst concentrations in Fig 3. The initial rates for Hoechst accumulation in the cytoplasm were calculated as *V*_*I*_ *= A*_*2*_*∙k* and plotted in Fig 3C.

To derive the kinetic parameters, the experimental initial rates and steady state concentrations were fit to Eqs 9 and 14. To this end, the equations were arranged to the following form:

$$I=\frac{1}{2}∙\left[-\frac{Kmc}{1+B}(K_{E}+1)-\frac{B-1}{1+B}∙\frac{O}{K_{c}}+\sqrt{{\left(\frac{Kmc}{1+B}(K_{E}+1)+\frac{B-1}{1+B}∙\frac{O}{K_{c}}\right)}^{2}+4\frac{Kmc}{1+B}∙\frac{O}{K_{c}}}\right],$$ (15A)

$$V_{I}=\frac{1}{2}∙\left[X_{v}-k_{m}-k_{v}+\sqrt{{\left(X_{v}-k_{m}-k_{v}\right)}^{2}+4k_{m}X_{v}}\right],$$ (15B)

$$k_{m}=k_{3}∙Km,$$ (15C)

$$k_{v}=\frac{V}{1+r∙(1-\varphi )},$$ (15D)

$$X_{v}=\frac{F\varphi }{1+r∙(1-\varphi )},$$ (15E)

$$\varphi =\frac{O}{O+2K_{D}},$$ (15F)

where *r = k*_*2*_*/k*_*3*_, *K*_*c*_ *= k*_*2*_*/k*_*1*_*∙k*_*4*_*/k*_*3*_ is the equilibrium constant for Hoechst transfer from cytoplasm into the medium, and *Kmc = Km∙k*_*3*_*/k*_*4*_. In addition, we introduced a parameter ω, which accounts for the flux increase upon induction of FhuA: *F*_*WT-Pore*_ *= ω∙F*_*WT*_. The data for *A*_*2*_ and *V*_*I*_ were fit to, respectively, Eqs 15A and 15B together, simultaneously for the WT and WT-Pore cells using the same set of adjustable parameters. Parameters *F*, *r*, *Kmc*, *K*_*E*_, *B*, *K*_*c*_, *ω* and *K*_*D*,*WT-Pore*_ were treated as independent, whereas the rest were expressed through them using constraints imposed by the model. The best fit values were: ω = 1.33, *K*_*c*_ = 0.45, *Kmc* = 0.0052 μM, *K*_*E*_ = 420, *B* = 0.98, *B*_*WT-Pore*_ = *B/ω* = 0.74, *F* = 0.008 μM/s, *r* = 0.44, *K*_*D*,*WT*_ = 1.0 μM, *K*_*D*,*WT-Pore*_ = 0.5 μM.

*A*_*1*_ values were fit to the model using Eq 14B and by postulating a limited number of Hoechst binding sites in the periplasm. The latter conclusion arises from the finding that *A*_*1*_ and *V*_*I*_ values are linearly related at low intracellular drug concentrations (S5C Fig), as predicted by Eq 14, but deviate from linearity at high intracellular drug levels (S5D Fig). Since the observed enhancement in fluorescence occurs only due to Hoechst binding to its target, such saturating behavior indicates that the signal is limited by the availability of the periplasmic target of the drug. Using the Langmuir binding model, the amount of the target-bound Hoechst can be calculated as

$$A_{1}=T_{0}∙\frac{P}{P+K_{T}}=T_{0}∙\frac{V_{I}}{V_{I}+{k_{3}K}_{T}},$$ (16)

where *T*_*0*_ is the amount of the Hoechst binding partner in the periplasm, *K*_*T*_ is the dissociation constant for the binding, and the second half of the equation incorporates Eq 14B. Eq 16 contains only two adjustable parameters, *T*_*0*_ and *k*_*3*_*K*_*T*_. Their best fit values (Fig 3E) were *k*_*3*_*K*_*T*_ = 0.9 nM/s, *T*_*0*_ = 1.0 μM.

### Strains and microbiological assays

All *E*. *coli* strains used in this study are derivatives of BW25113 (WT strain Δ(araD-araB)567 Δ(rhaD-rhaB)568 ΔlacZ4787 (::rrnB-3) hsdR514 rph-1) [49]. The ΔTolC derivative (BW25113 Δ*tolC-ygiBC*) lacks an outer membrane channel TolC, which is required for activities of at least nine different exporters, including the major efflux pump AcrAB-TolC [22, 50]. Inactivation of TolC dramatically increases susceptibility of *E*. *coli* cells to a variety of antimicrobial agents. The gene encoding *fhuA ΔC/4L* (the Pore) under the control of the arabinose inducible promoter P_BAD_ was inserted at the respective *att*Tn7 site downstream of the *glm*S gene to construct WT-Pore and ΔTolC-Pore strains [46].

To measure IC_50_, exponentially growing cells were inoculated at a density of 10^5^ cells per ml into wells containing LB broth (10 g/L tryptone, 5 g/L yeast extract, 5 g/L NaCl) supplemented with 0.1% arabinose and a serially diluted drug in question, and incubated for 18h at 37°C. Cell growth was then determined by measuring the absorbance at 600 nm using Tecan Spark 10M multimode microplate reader.

### Fluorescence and protein assays

Fluorescence of free Hoechst and its DNA- and lipid-bound forms was analyzed first for calibration purposes. Hoechst solutions were mixed with different amounts of salmon sperm DNA (Invitrogen Inc.) in buffer containing 50 mM HEPES-KOH (pH 7.0), 1 mM MgSO_4_ and 0.4% glucose (HMG buffer). Both direct and serial dilutions were prepared in a black F-bottom non-binding 96-well plate (Greiner Bio-One, Inc.). OD_600_ 1.0 of *E*. *coli* cells was assumed to contain 17 μg of DNA [51]. In parallel, the corresponding solutions of free Hoechst in HMG buffer were prepared. The plates containing DNA-bound and free Hoechst were incubated for different periods of time up to 120 min and emission spectra were collected in a Tecan Spark 10M plate reader at λ_ex_ = 355nm and λ_em_ from 400 nm to 550 nm with a Z-value setting of 25000 and a gain of 75. At these conditions, fluorescence of free Hoechst was found to have emission maximum at λ_em_ = 500 nm and its DNA-bound form at λ_em_ = 450 nm and the intensity remained the same for up to 120 min. The signal-response curve for the reader was found linear over the entire range of measurements.

Similar calibrations were carried out with purified *E*.*coli* lipids (a polar fraction, Avanti Lipids Inc.) and lysed ΔTolC-Pore cells. Dry lipids were reconstituted in HMG buffer and briefly sonicated in a water bath sonicator (Branson Inc.). The lipid suspension containing 27 μg, 13.5 μg and 2.7 μg of lipids, which correspond to lipid contents of *E*. *coli* cells at OD_600_ of 1.0, 0.5, 0.1 [51] was mixed with Hoechst solutions as described for DNA calibrations. Cell lysates were prepared by sonication and freeze-thawing of exponentially grown ΔTolC-Pore cells collected by centrifugation and resuspended in HMG buffer to OD_600_ 2.0. Lysates were mixed with Hoechst solutions to obtain final amounts of lysed cells corresponding to OD_600_ of 1.0, 0.5, 0.25 and 0.1.

For Hoechst uptake experiments, overnight grown cells were re-inoculated into 30 ml LB broth and grown until OD_600_ of 0.3, induced with 0.1% arabinose and grown until OD_600_ of 1. The protocol was somewhat modified for arabinose titration experiments, wherein the overnight culture was diluted to OD_600_ of 0.03, supplemented with 0.1% glucose to suppress the leaky expression of FhuA*, and grown up to OD_600_ of 0.3. The cells were then washed, resuspended in fresh LB broth containing indicated concentrations of arabinose and grown until OD_600_ of 1. For both protocols, the cells were then pelleted by centrifugation for 15 min at room temperature and washed once in HMG buffer. The pellet was resuspended in HMG buffer to OD_600_ of 2.0 and kept at room temperature during the course of the experiment. For the uptake kinetics, 100 μl of Hoechst solution in HMG buffer at twice the concentrations indicated was taken into a black F-bottom non-binding 96-well plate. Fluorescence of Hoechst was followed at λ_ex_ = 355 nm and λ_em_ = 450 nm. To this, 100 μl of cells at OD_600_ of 2.0 was injected, shaken for 6 sec and the fluorescence of Hoechst was monitored continuously for up to 10 min. Fluorescence was read every 10–20 sec with shaking prior to the readings. All measurements were done at least in triplicates. Fluorescence microscopy was done as previously described [52].

To measure the amounts of FhuA* in cellular membranes, cells were induced at indicated concentrations of arabinose as described above. Membrane fractions were isolated and quantitative immunoblotting with a monoclonal anti-His antibody (Sigma) was carried out as described before [46].

## Supporting information

S1 Fig Graphic analysis of the *R*-function. The *R*-function describes uptake behavior by cells with active efflux and provides a steady state solution to a class of differential equations. (**a**) Asymptotic behavior of the *R*-function. (**b-d**) The effect of parameters *a*, *b* and *c* on the shape of the *R*-function. Note that a change in the sign of *c* leads to a phase transition from concave to convex shape of the *R*-versus-*X* dependence.
(PDF)

S2 Fig Modeling drug accumulation in bacteria. (**a**) A typical simulated time course of drug uptake obtained by numeric integration of Eq 1 using the following parameters: *k*_*1*_ = *k*_*2*_ = *k*_*3*_ = 1.25; *k*_*4*_ = 0.01 (to recuperate the existence of the slow phase in drug uptake); *k*_*5*_ = *k*_*6*_ = 0.125; *Km* = 0.0024; *V* = 1; *O* = 30. These values were chosen to match simulations in Fig 2C (*B* = 0.8; *K*_*E*_*’* = 167; *K*_*D*_ = 1). Shown are drug concentrations in the periplasm (*P*) and cytoplasm (*I*) and the fractional saturation *φ* of the outer membrane barrier (*M*). The initial rates of drug permeation into the cytoplasm *V*_*I*_ can be determined by fitting post-lag data for *I* to a linear trend line. (**b**). A comparison of the initial rates of drug build-up in the cytoplasm using numeric integration as described in panel **a** (Numeric) and Eq 5 (*R*-function).
(PDF)

S3 Fig Hoechst fluorescence spectra. Fluorescence emission spectra for 4 μM Hoechst 33342 (HT) alone, or in complex with either DNA or the *E*. *coli* total lipids (polar fraction).
(PDF)

S4 Fig Uptake of Hoechst 33342 (HT) by WT and WT-Pore cells. (**a, b**) Time courses of HT uptake (±SD; *n* = 4) for WT (**a**) and WT-Pore (**b**) cells at the indicated HT concentrations (in μM). Lines show the fit to the burst-single exponential decay as described in Fig 3A. (**c, d**) A correlation between the steady state HT concentration in the periplasm (determined as the best fit *A1* value in panels **a** and **b**) and the initial rates of the cytoplasmic HT build up that are shown in Fig 3C. See Methods for details on fitting.
(PDF)

S5 Fig Uptake of Hoechst 33342 (HT) by WT-Pore cells at various arabinose concentrations. (**a, b**) The initial rate (**a**) and steady state accumulation (**b**) of cytoplasmic Hoechst. The two data sets were fit to Eq 15 by adjusting the values of *B* while keeping the rest of the parameters at their best-fit values determined in Fig 3. Note the higher best-fit values of *B*, which are caused by the use of glucose in this experiment resulting in better suppression of FhuA* leakage and its higher relative induction by arabinose.
(PDF)
