# Supplementary figures and images for: Bifurcation kinetics of drug uptake by Gram-negative bacteria
Source: PLoS One. 2017 Sep 19;12(9):e0184671. doi: 10.1371/journal.pone.0184671 (PMC5604995; doi:10.1371/journal.pone.0184671)

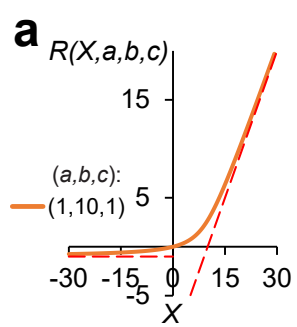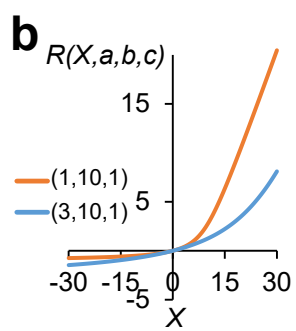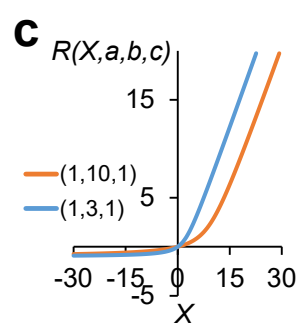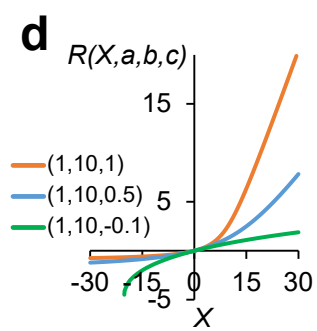

Supplement: S1 Fig — The R-function describes uptake behavior by cells with active efflux and provides a steady state solution to a class of differential equations. (a) Asymptotic behavior of the R-function. (b-d) The effect of parameters a, b and c on the shape of the R-function. Note that a change in the sign of c leads to a phase transition from concave to convex shape of the R-versus-X dependence. (PDF) [file pone.0184671.s001.pdf]

**a**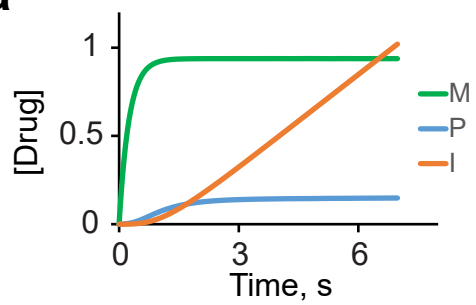**b**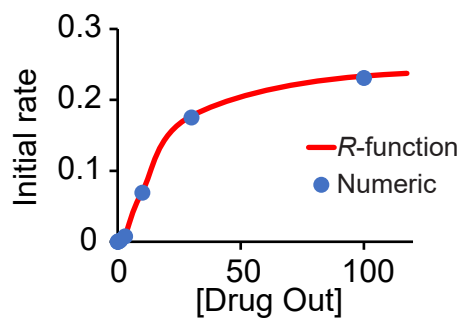

Supplement: S2 Fig — (a) A typical simulated time course of drug uptake obtained by numeric integration of Eq 1 using the following parameters: k1 = k2 = k3 = 1.25; k4 = 0.01 (to recuperate the existence of the slow phase in drug uptake); k5 = k6 = 0.125; Km = 0.0024; V = 1; O = 30. These values were chosen to match simulations in Fig 2C (B = 0.8; KE’ = 167; KD = 1). Shown are drug concentrations in the periplasm (P) and cytoplasm (I) and the fractional saturation φ of the outer membrane barrier (M). The initial rates of drug permeation into the cytoplasm VI can be determined by fitting post-lag data for I to a linear trend line. (b). A comparison of the initial rates of drug build-up in the cytoplasm using numeric integration as described in panel a (Numeric) and Eq 5 (R-function). (PDF) [file pone.0184671.s002.pdf]

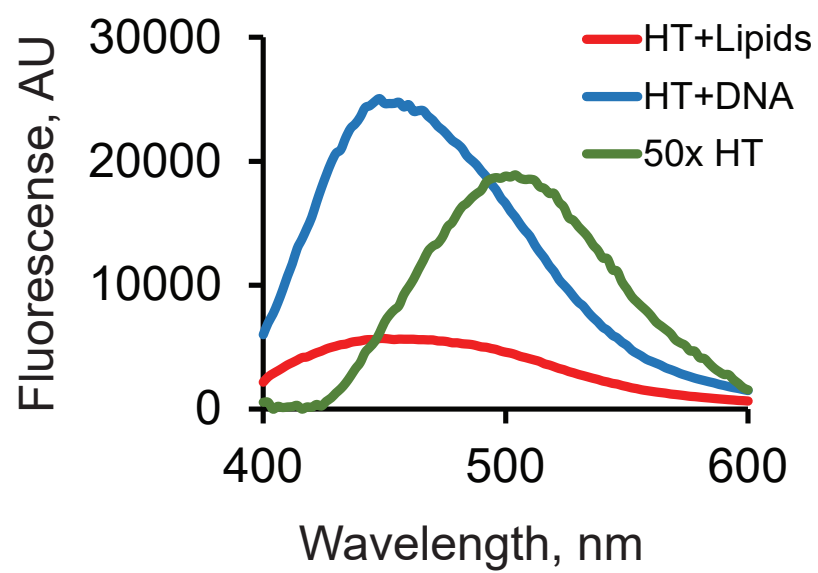

Supplement: S3 Fig — Fluorescence emission spectra for 4 μM Hoechst 33342 (HT) alone, or in complex with either DNA or the E. coli total lipids (polar fraction). (PDF) [file pone.0184671.s003.pdf]

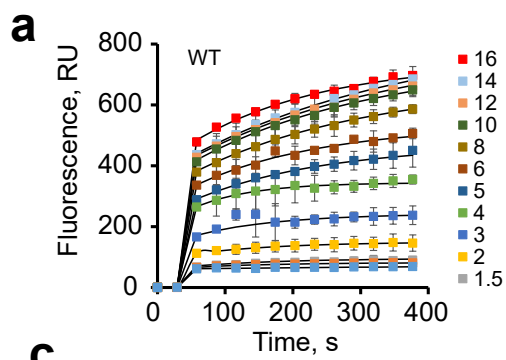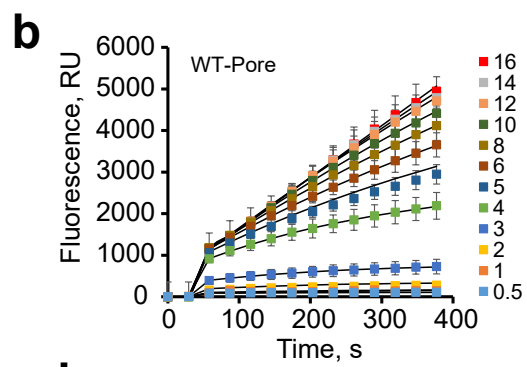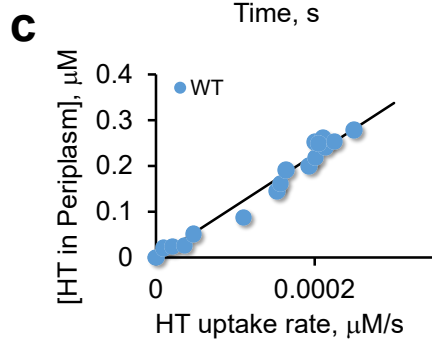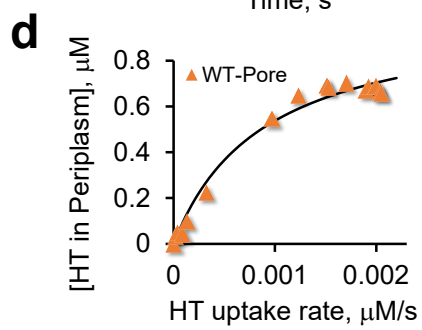

Supplement: S4 Fig — (a, b) Time courses of HT uptake (±SD; n = 4) for WT (a) and WT-Pore (b) cells at the indicated HT concentrations (in μM). Lines show the fit to the burst-single exponential decay as described in Fig 3A. (c, d) A correlation between the steady state HT concentration in the periplasm (determined as the best fit A1 value in panels a and b) and the initial rates of the cytoplasmic HT build up that are shown in Fig 3C. See Methods for details on fitting. (PDF) [file pone.0184671.s004.pdf]

**a**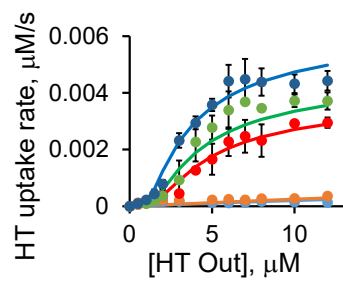**b**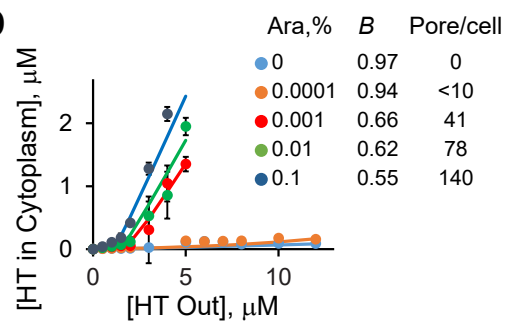

Supplement: S5 Fig — (a, b) The initial rate (a) and steady state accumulation (b) of cytoplasmic Hoechst. The two data sets were fit to Eq 15 by adjusting the values of B while keeping the rest of the parameters at their best-fit values determined in Fig 3. Note the higher best-fit values of B, which are caused by the use of glucose in this experiment resulting in better suppression of FhuA* leakage and its higher relative induction by arabinose. (PDF) [file pone.0184671.s005.pdf]
